# Cancer incidence data at the ZIP Code Tabulation Area level in the United States interpolated by Monte Carlo simulation with multiple constraints

**DOI:** 10.1038/s41597-025-05254-8

**Published:** 2025-05-30

**Authors:** Lingbo Liu, Fahui Wang, Tracy Onega

**Affiliations:** 1 Center for Geographic Analysis, Harvard University, Cambridge, MA USA; 2 Department of Geography and Anthropology, Louisiana State University, Baton Rouge, LA USA; 3 Huntsman Cancer Institute, University of Utah, Salt Lake City, UT USA; 4 Department of Population Health Sciences, University of Utah, Salt Lake City, UT USA

**Keywords:** Health care, Environmental social sciences

## Abstract

High-quality cancer data are fundamental for public health research and policy, but cancer data for small geographic units and population subgroups in the United States are rarely available due to small-sample suppression rules, spatial coarsening, and data incompleteness. These limitations hinder high-resolution spatial analyses and precision public health interventions. This study provides a high-resolution cancer incidence dataset for the U.S., generated through a multi-constraint Monte Carlo simulation framework that reconstructs suppressed county-level cancer data and systematically disaggregates them to ZIP Code Tabulation Areas (ZCTAs), guided by demographic constraints. This method integrates population subgroup structures and macro-level incidence rates as constraints, ensuring consistency and reliability across spatial scales. The resulting dataset spans multiple geographic units, from state and county levels to ZCTAs, enabling comprehensive analyses of cancer burden, in-depth spatial analyses, and precision public health interventions across multiple scales.

## Background & Summary

Cancer data serve as a critical foundation for monitoring cancer burden, supporting health geography research and informing public health policy^1,2^. However, existing datasets have significant limitations in terms of completeness, spatial resolution, and usability due to privacy protection regulations and small-sample suppression rules^3,4^. These challenges not only compromise the accuracy of disease burden assessments but also hinder precise interventions and resource optimization for specific populations and small geographic areas^5^. Particularly within the current context of precision public health, there is an urgent need for high-resolution, structurally consistent, and transparent cancer data that can support in-depth spatial analysis of disease distribution and scientific allocation of health care resources^6^. The U.S. National Cancer Institute (NCI) provides publicly available cancer data through the State Cancer Profiles. However, these datasets are restricted to the county level and are subject to extensive data suppression and missing values, implemented to safeguard privacy^7^. This not only impedes a precise understanding of disease risks but also creates data bottlenecks for targeted public health interventions^8^. While cancer data are widely collected and applied at the county level and above, studies at finer spatial units remain a rarity. As scientific research increasingly demands data openness and reproducibility, developing a high-resolution and transparent cancer data framework has become a critical issue.

Various methods, such as multiple imputation (MI)^9,10^, geo-imputation^11,12^, dasymetric mapping^13–16^, geostatistical interpolation^17^, and machine learning-based approaches^18,19^, have been employed for missing data handling and data downscaling. However, these approaches have not yet fully leveraged multi-level nested structures while adhering to total quantity constraints^20^. This limitation remains a significant obstacle to conducting high-accuracy, multi-scale analyses of cancer burden using openly accessible data^21^.

**Fig. 1:**
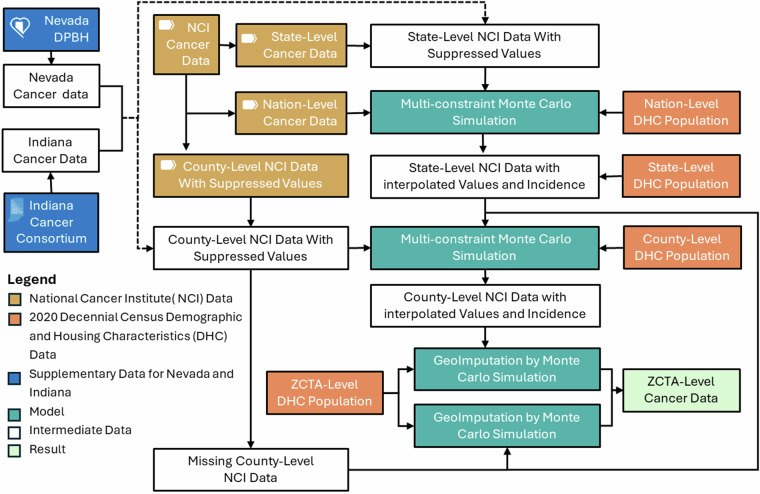
Workflow for constructing the dataset.

This study employs a multi-constrained Monte Carlo simulation approach to integrate population subgroup structures and macro-level cancer incidence rates as constraints^22,23^ and uses probabilistic simulation techniques to interpolate suppressed cancer data (i.e., annual counts ≤ 3 over 5 years, withheld under NCI privacy rules). A pilot study in Utah validated this method,  demonstrating high precision and consistency across different spatial scales and population groups^24^. This national-level study builds on that pilot work by extending the method to cover all U.S. states, addressing missing and suppressed values at both the state and county levels. While the core methodology remains consistent, the technical descriptions have been rewritten to reflect the generalized implementation and expanded spatial coverage. Here, the method is used to expand the scope by first estimating missing or suppressed cancer data at the state and county level and further downscale it to the ZIP Code Tabulation Area (ZCTA) in the United States. ZCTAs are approximations of U.S. Postal Service ZIP codes, corresponding roughly to between administrative levels II and III. They are defined by the U.S. Census Bureau based on residential addresses and population distributions, and their boundaries may change over time with decennial census updates. The resulting dataset spans multiple spatial scales (e.g., state, county, and ZCTA) and demographic subgroups stratified by age, sex, and race/ethnicity, providing robust data support for analysing the spatial distribution of cancer risk^25^, urban-rural disparities^26^, and the effects of population structures^27^. In regions with extensive data suppression, these high-resolution data enable more accurate disease burden assessments and optimized allocation of health care resources such as mobile cancer screening units, targeted outreach programs, and localized public health interventions. The method could be extended to finer spatial units, such as census tracts or blocks, though smaller subgroup populations at these scales may limit estimation reliability. Figure 1 illustrates the workflow.

A major innovation of this dataset lies in its capability of multi-level integration. By interpolating suppressed data at the state and county level and downscaling to ZCTA, this approach incorporates multi-level total quantity constraints^28^, accounts for geographical heterogeneity across states and counties^29^, and leverages the latent continuity of Average Annual Percent Change (AAPC) across population, race, gender, and age groups^30^. This integration bridges the analytical gap between micro-level data and macro-level trends, providing a robust and comprehensive tool for health research across varying scales. At the macro level, researchers can leverage statistical models to reveal spatial patterns of cancer distribution and explore the effects of social, economic, and environmental factors on disease risks. At the micro level, the combination of high-resolution population data and health indicators enables finer-grained risk assessments and causal analyses, offering a scientific basis for public health policymaking.

By publicly releasing this high-resolution, multi-scale cancer dataset for the U.S., we aim to provide high-quality, reproducible data support for researchers, policymakers, and practitioners in the fields of health geography and public health. This effort advances the scientific development of cancer risk assessments and precision health interventions while meeting global standards for data openness and transparency.

## Methods

### Data sources

#### NCI Cancer data at the national, state and county levels

**Fig. 2:**
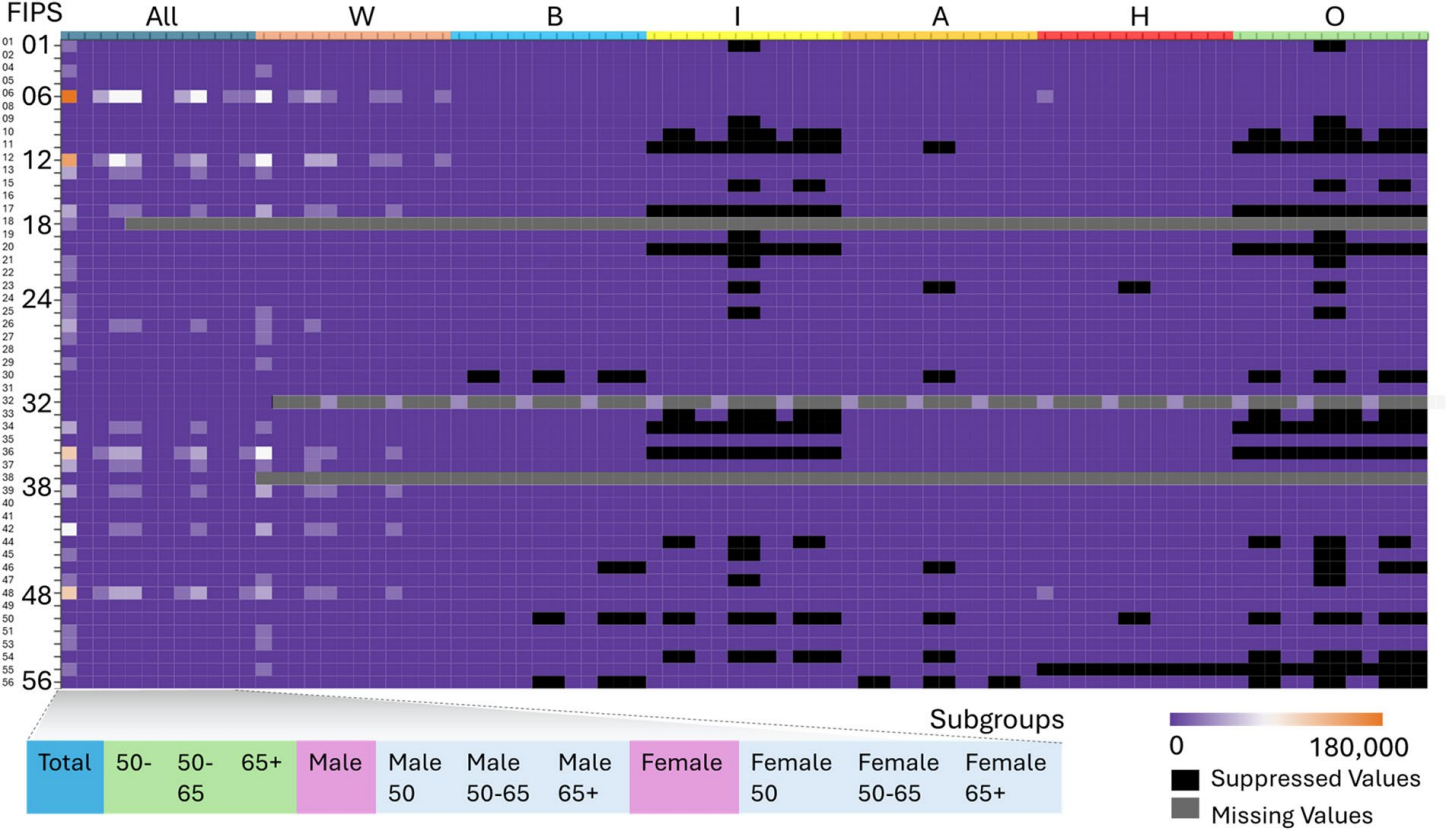
Heatmap of reported and suppressed cancer incidence data across U.S. states and demographic subgroups (2016–2020). Each row on the y-axis corresponds to a U.S. state, identified by its FIPS state code (1–56, including DC and territories). Each column on the x-axis represents a race/ethnicity group (All, W: White, B: Black, I: American Indian, A: Asian, H: Hispanic, O: Other), subdivided by age-sex groups (e.g., Male 50–65, Female 65+), forming the 36 population subgroups used in this study. Cell colors indicate the reported average annual cancer case counts (from 0 to 180,000, purple to orange): Black cells indicate suppressed values (i.e., counts ≤ 3) that were masked due to NCI privacy protection policies; Gray cells indicate missing values not reported at all for that subgroup in that state. Color-coded subgroup categories at the bottom denote combinations of age and sex used in the stratified modeling framework.

Our study relies on cancer incidence data provided by the National Cancer Institute (NCI), accessible through the State Cancer profiles platform (https://statecancerprofiles.cancer.gov). These data are collected by the National Program of Cancer Registries and SEER, reported by individual state cancer registries, and harmonized by the National Cancer Institute for public release. The most recent dataset spans 2016–2020 and includes annual average cancer incidence counts categorized by age group, sex, and race/ethnicity at both state and county levels. 36 subgroups were derived from 6 racial/ethnic groups (All; Non-Hispanic White, Non-Hispanic Black, Non-Hispanic American Indian and Alaska Native, Non-Hispanic Asian and Pacific Islander, Hispanic, and Other), 2 sexes (female, male), and 3 age groups (<50, 50–65, >65). Certain counties report suppressed annual average values (≤3 cases) due to privacy protection rules, while some states and counties completely lack reported data. Figure 2 presents the NCI Cancer Data at the county level, including the total cancer counts across various racial-ethnic groups.

#### Supplementary data for Nevada and Indiana at the state and county levels

As cancer data for Nevada and Indiana were entirely missing from the NCI State Cancer Profiles database, we included supplementary datasets to ensure complete national coverage. Data for Nevada (2020) were provided by the Nevada Department of Public and Behavioral Health (DPBH), including state-level cancer incidence counts data stratified by age and race, and county-level data for Clark and Washoe counties by sex (https://dpbh.nv.gov/uploadedFiles/dpbh.nv.gov/content/Programs/NCCR/dta/Incidence%20Rates%20of%20leading%20Cancers_2020.pdf). Indiana data (2013–2017) is from the Indiana Cancer Consortium^31^, which includes overall state-level cancer counts by age groups and total county-level cancer data (https://indianacancer.org/wp-content/uploads/2021/10/ICC_FF_Intro_2021.pdf). These datasets precede or lag behind the NCI data window (2016–2020) but serve as the most accurate supplementary sources available to the authors’ knowledge.

#### US Census population data

Population data were extracted from the 2020 Decennial Census Demographic and Housing Characteristics (DHC) at nation, state, county, and ZIP Code Tabulation Area (ZCTA) levels (https://www.census.gov/data/tables/2023/dec/2020-census-dhc.html). The 2020 census data were used as a consistent demographic baseline for the 2016–2020 cancer incidence period, assuming subgroup proportions remain relatively stable over the short term. Aligned with the NCI cancer data, the population data were aggregated into 36 subgroups based on age, sex, and race/ethnicity. For ZCTAs spanning multiple counties, census block-level data were used to proportionally assign population counts.

### Data imputation and downscaling by monte carlo simulation

The overall framework in Fig. 1 outlines a stepwise approach encompassing the imputation of suppressed or missing values across varying geographic levels and the subsequent downscaling of data to ZCTAs. This process involves employing Multi-constraint Monte Carlo (MMC) and applying Geo-Imputation techniques. The former is primarily used to address suppressed or missing values at the state and county levels, while the latter is used to downscale data from state or county units to the ZCTA level.

#### Multi-constraint Monte Carlo (MMC) for suppressed county-level data

To impute suppressed cancer counts at the county level, we implemented a Monte Carlo simulation framework governed by multiple hierarchical constraints. The model relies on 36 subgroup-specific population structures at the county scale and aligns simulated counts with state-level incidence rates benchmarks. This method follows the simulation logic illustrated in Fig. 5 of our prior study^24^. The procedure unfolds through four distinct stages:

- **Multiple Constraint initialization**: For each state, and for each racial/ethnic group, we constructed a k × 6 matrix for its k counties, where the six columns correspond to fine-grained demographic splits by age and sex (Male <50, Male 50–65, Male 65+, Female <50, Female 50–65, Female 65+). Row constraints were set based on broader demographic totals at the county level, such as male, female, age <50, age 50–65, age ≥65, and total. Column constraints were set based on state-level subgroup-specific cancer incidence counts, ensuring consistency between county distributions and aggregated state totals.
- **Probabilistic Case Allocation**: Using the proportional distribution of the county-level subgroup populations and the state-level subgroup incidence rates, we constructed an allocation probability for each matrix cell. During the simulation, one case is randomly assigned to a subgroup cell according to this probability. After assignment, both the corresponding row (county total constraint) and column (state subgroup constraint) are decremented by one. When any constraint (row or column) reaches zero, the corresponding cells are locked, and no further cases are allocated to them. This process continues iteratively until all constraints are satisfied, ensuring that both county- and state-level cancer case totals are respected.
- **Iteration and optimization**: To address the stochastic nature of the Monte Carlo simulation, the process was repeated 1,000 times independently. The final imputation result was selected as the simulation instance with the smallest overall variance from the average distribution across iterations, ensuring stability and robustness.
- **Hierarchical estimation**: For states where certain county-level totals were missing (e.g., missing overall totals or subgroup totals), a similar MMC was implemented. In these cases, a k × 7 matrix was constructed, with seven columns representing broad race/ethnicity groups (White, Black, American Indian/Alaska Native, Asian, Hispanic, Other) and one column for total counts. Similar population-proportional and incidence rate-informed Monte Carlo sampling was then applied to impute missing entries, following the same constraint satisfaction principles.

#### Geo-Imputation for ZCTA-level subgroup data

To further disaggregate county-level estimates to the ZIP Code Tabulation Area (ZCTA) level, we adapted a stochastic imputation approach that maps subgroup-level case distributions using fine-scale population structures. This approach employs a binning-based assignment strategy derived from cumulative population shares.

- **Stochastic matrix generation:** For each race/ethnicity subgroup and its corresponding age-sex breakdown (six fine subgroups), the total number of cancer cases m in each county was identified. A stochastic matrix of dimensions m × n was generated, where each cell contained a random number drawn from a uniform distribution between 0 and 1. Each column represents one independent simulation of case allocation.
- **Population-based cumulative share binning:** The population distribution of the given subgroup across all ZCTAs within the county was used to compute cumulative shares. Each ZCTA was assigned a continuous probability interval based on its cumulative subgroup population proportion. ZCTAs with larger subgroup populations occupied larger bins, increasing their likelihood of receiving assigned cases.
- **Simulated count allocation**: For each random value in the stochastic matrix, a ZCTA bin was identified according to which cumulative interval the value fell into. This probabilistic assignment ensures that cancer cases are distributed proportionally to the spatial distribution of the subgroup population across ZCTAs.
- **Result optimization**: Among all simulation iterations, the optimal result was identified as the one with the lowest standard deviation from the ensemble mean across all ZCTAs. This approach balances statistical representativeness and integrity with respect to total counts and subgroup allocations.

For the 1,000 independently seeded Monte Carlo simulations toward robust estimation, the mean across all simulations offers an intuitive approximation of subgroup distributions^32^. However, it typically produces non-integer values that conflict with the discrete nature of case counts—especially in small populations or under suppression thresholds. Therefore, we selected the optimal scenario by identifying the simulation run with the minimum standard deviation from the overall distribution^33,34^, i.e., the simulation with the minimum standard deviation from the means indicates the closest alignment to the average pattern and is thus the best representation of the possible subgroup distribution.

### Major data processing steps

#### State-level cancer data interpolation

**Fig. 3:**
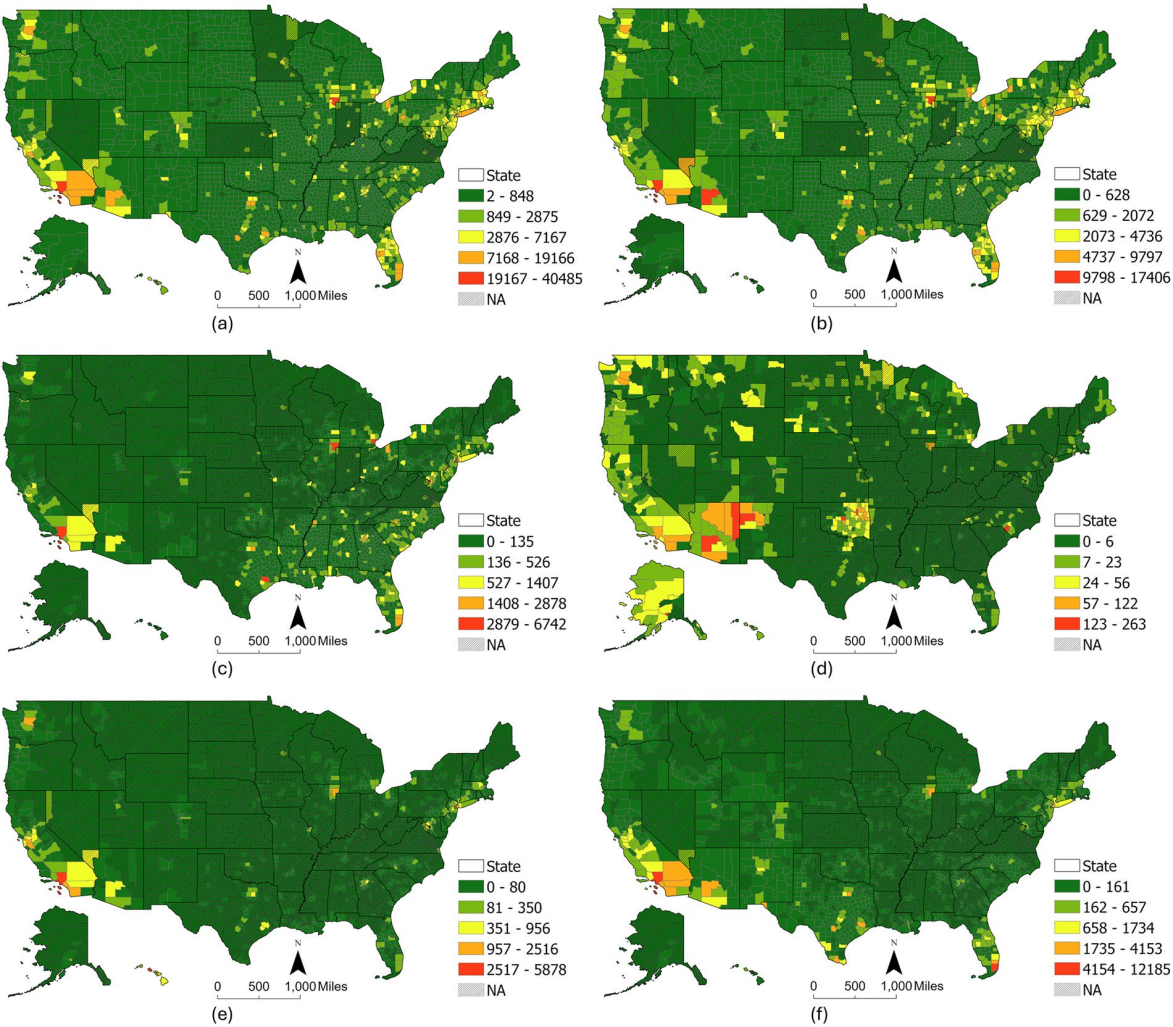
Interpolated cancer incidence counts at the county level: (**a**) All Race including Hispanic, (**b**) Non-Hispanic White, (**c**) Non-Hispanic Black, (**d**) Non-Hispanic American Indian and Alaska Native, (**e**) Non-Hispanic Asian and Pacific Islander, (**f**) Hispanic. NA represents the missing values in the original NCI dataset.

Among the 51 state-level units (including DC), 25 had no missing values, while 26 states contained suppressed or missing data (minimum missing entries 4, maximum 80, and average 20), as shown in Fig. 3. National-level subgroup population, incidence rates, and state-level population subgroup data were utilized to estimate suppressed or missing state cancer data by the Multi-constraint Monte Carlo (MMC) simulation. Subgroup incidence rates at the state level were calculated hierarchically and used as input for county-level imputation.

#### County-level cancer data interpolation

For states with at least one county having reported total cancer counts, the county-level cancer data interpolation was applied. Of the 3,142 counties in the NCI cancer data and 3,143 in the DHC population data, 3,140 matched; the two unmatched NCI counties were assigned state-level incidence rates. The state level subgroup cancer incidence rates, county level subgroup population and cancer data were utilized to implement the Multi-constraint Monte Carlo (MMC) simulation method. Figure 3 shows the interpolated county level cancer data.

#### Geo-imputation from state/county to ZCTA

The NCI county-level cancer data, in combination with interpolated data for missing or suppressed entries, were utilized by the Geo-Imputation method to generate the ZCTA-level cancer data based on their population structures. For states with missing county-level cancer data (e.g., Indiana, Kansas, Minnesota, Nevada, Virginia), direct state-to-ZCTA imputation was performed by the Geo-Imputation method to directly allocate state-level cancer counts proportionally to ZCTAs.

#### Final integration

**Fig. 4:**
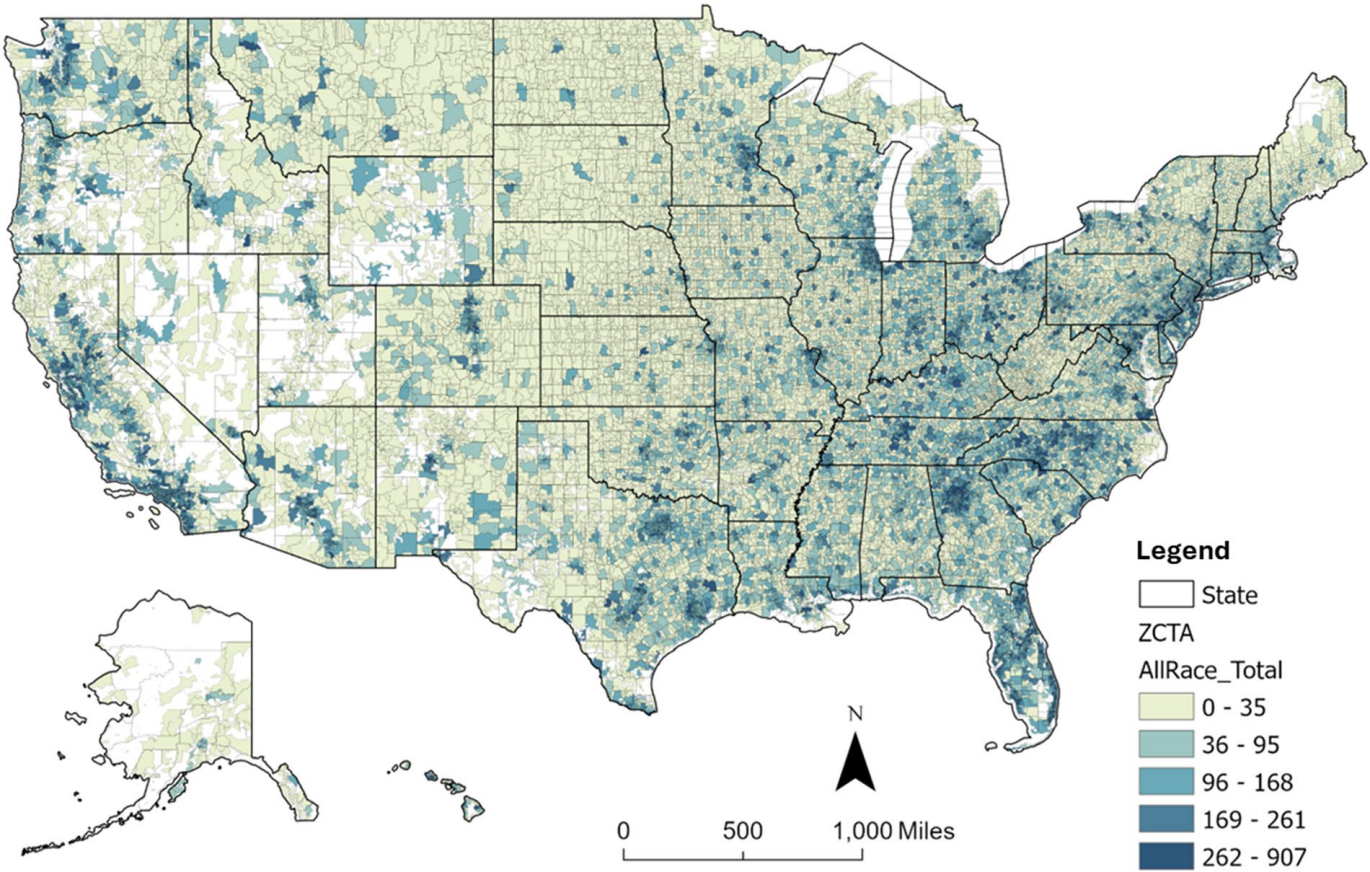
Interpolated total cancer incidence counts at the ZCTA level.

ZCTA-level subgroup cancer counts were aggregated across all counties and states, ensuring consistency in overlapping ZCTAs across counties and states. For ZCTAs spanning multiple counties or states, cancer cases were merged proportionally based on population distributions. The complete ZCTA-level cancer dataset integrates subgroup cancer incidence counts data across all spatial levels, as shown in Fig. 4.

## Data Records

The dataset generated and analysed in this study is publicly available in Harvard Dataverse: 10.7910/DVN/W3S2LW^35^. The dataset is structured to facilitate its use in various applications, including health geography studies, spatial epidemiology, resource allocation, and public health research reports. The dataset is organized into clearly labelled subfolders:

### Raw NCI CSV tables

This category contains the original 2016–2020 cancer incidence data from the National Cancer Institute (NCI) SEER platform.

- **NCI_Nation_Original.csv:** National-level total cancer counts, excluding Nevada and Indiana due to data unavailability.

- **NCI_State_Original.csv:** State-level subgroup-specific cancer incidence counts (by age, sex, and race/ethnicity) from the NCI State Cancer Profiles, with suppressed values where privacy rules apply.
- **NCI_County_Original.csv:** County-level subgroup-specific cancer incidence counts, including suppressed and missing values.

### Processed and result tables

These tables include the outputs from the multi-constraint Monte Carlo simulation and geo-imputation processes, which address suppressed and missing values while downscaling data to finer spatial units.

- **NCI_State_Harmonized.csv:** Harmonized state-level cancer counts, incorporating supplementary data for Nevada and Indiana.
- **NCI_State_Imputed.csv:** Interpolated state-level subgroup-specific cancer incidence counts, with suppressed values imputed using the MMC method.
- **NCI_State_Incidence_Imputed.csv:** Estimated subgroup-specific incidence rates at the state level, derived by dividing cancer counts by corresponding subgroup population sizes.
- **NCI_County_Harmonized.tab:** Harmonized county-level cancer counts, integrating Nevada and Indiana supplementary data and resolving county definition discrepancies.
- **NCI_County_Interpolated.tab:** Interpolated county-level subgroup-specific cancer counts, with suppressed and missing values imputed via the MMC method.
- **NCI_ZCTA_Imputed.tab:** Interpolated ZIP Code Tabulation Area (ZCTA)-level cancer counts for all 36 demographic subgroups, generated using the geo-imputation process.

Although the dataset provides estimated incidence counts, incidence rates can be derived by dividing counts by the corresponding subgroup population sizes available from the 2020 Census DHC data

## Technical Validation

To ensure the accuracy and reliability of our dataset, we conducted a series of validation experiments at the state, county, and ZIP Code Tabulation Area (ZCTA) levels. These experiments evaluated the performance of the Multi-Constraint Monte Carlo (MMC) simulation and Geo-Imputation methods in addressing suppressed and missing values. A random masking validation approach was employed, wherein portions of known data were deliberately masked to simulate suppression or missingness. The MMC method was subsequently applied to predict the masked values, and the predictions were compared to the original values. This approach follows a 10-fold Monte Carlo Cross-Validation (MCCV) framework, providing a robust evaluation of interpolation methods through repeated masking and prediction iterations.

In addition to evaluating the MMC method, we compared its performance with baseline statistical and machine learning models, including Ordinary Least Squares (OLS), Random Forest (RF), and Extreme Gradient Boosting (XGBoost). For each test fold, cancer incidence counts were predicted using only subgroup-level demographic features, including age, sex, race/ethnicity compositions (e.g., AllRace, Non-Hispanic Black, Hispanic), and total population counts. All models used the same input variables to ensure a fair comparison. Evaluation was based on three commonly used metrics: Mean Absolute Error (MAE), Root Mean Squared Error (RMSE), and the coefficient of determination (R²). These metrics were selected due to their complementary strengths in assessing regression accuracy. MAE captures the average magnitude of prediction errors without considering direction; RMSE penalizes larger deviations more strongly by squaring the error terms; and R² quantifies the proportion of variance in observed data explained by the model. Together, these metrics provide a robust and interpretable framework for evaluating prediction performance, especially in small-area cancer incidence estimation involving smoothed numerical counts.

At the state level, we randomly selected 90% of U.S. states (n = 46) as training data and used the remaining 10% for testing. This process was repeated 10 times to ensure robustness. In each iteration, the MMC method estimated subgroup-specific cancer counts using state-level incidence rates and demographic distributions. As shown in Table 1, the MMC method outperformed the baselines across all metrics. On average, MMC achieved an R² of 0.997 (±0.002), MAE of 160.7 (±77.0), and RMSE of 363.0 (±197.0), demonstrating both high predictive accuracy and low variance across folds.

**Table 1:** 10-folded Cross-Validation at the State Level by all models.

	Metrics	Monte Carlo	OLS	Random Forest	XGBoost
R^2^	Mean	0.997	0.637	0.767	0.968
	Std	0.002	0.049	0.062	0.015
MAE	Mean	160.674	2361.427	1388.238	393.292
	Std	76.998	464.407	538.869	193.885
RMSE	Mean	362.954	4281.455	3553.127	1317.709
	Std	197.033	1589.665	1779.324	722.87

Note: mean absolute error (MAE), root mean squared error (RMSE), Std (Standard Deviation).

**Fig. 5:**
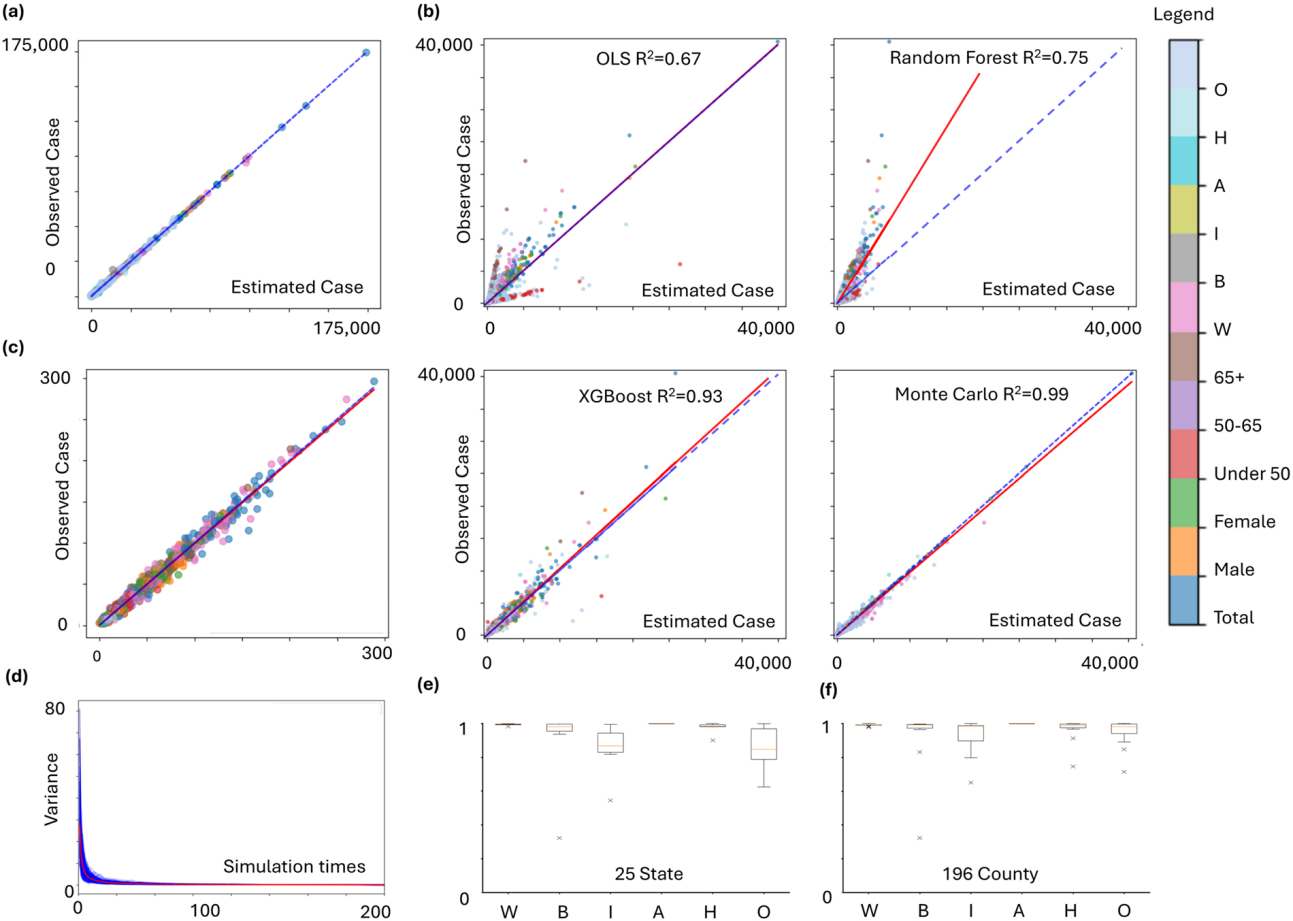
Validation of simulated data against observed data at different spatial levels at (**a**) State level (R² ≈ 1), (**b**) County level compared with 4 machine learning models (4 graphs), and (**c**) ZCTA level (R² = 0.98); (**d**) Variance trends across simulation iterations; Cross-validation on correlations for racial/ethnic groups at (**e**) the state level for 25 sampled states, and (**f**) the county level for 196 counties.

Figure 5d shows that after approximately 100 iterations, the variance stabilized, highlighting the robustness and stability of the approach.

At the county level, subgroup-specific cancer data for selected counties were randomly masked, while total values were retained. Using state-level incidence rates and county-level population distributions as constraints, the MMC method imputed the masked subgroup values. Validation focused on states with more than 30 counties and average missing total values below 60 (total *n* = 84), resulting in 25 selected states. For each state, 10% of subgroup-specific data (excluding “All Race”) were masked, predicted using the MMC workflow, and evaluated.

As shown in Table 2, MMC outperformed all baselines, achieving an average R² of 0.973, MAE of 19.6, and RMSE of 57.2. While XGBoost and OLS achieved relatively strong overall R², both exhibited instances of invalid predictions, including negative case counts, which are not plausible in this context. Figure 5b visualized the predictions of each model using scatterplots of observed versus estimated values. The MMC model displays the tightest alignment to the 1:1 line with minimal dispersion, indicating low bias and high precision. In contrast, Random Forest shows a systematic deviation from the diagonal, while OLS and XGBoost yield implausible negative values in some instances.

**Table 2:** 10-folded Cross-Validation at the County Level by all models.

	Metrics	MC	OLS	RF	XGBoost
R^2^	Mean	0.973	0.586	0.570	0.963
	Std	0.017	0.219	0.144	0.023
MAE	Mean	19.573	117.803	113.629	24.802
	Std	11.444	55.537	55.821	21.253
RMSE	Mean	57.225	246.896	264.412	88.808
	Std	36.237	208.926	239.656	115.449
Target vs Prediction	Min (0)	0.0	−261.095	7.126	−281.824
	Max (40485)	40485	39954	7180	25903
	Mean (160.967)	160.791	159.019	158.761	159.294
	Std (563.963)	579.149	460.197	269.754	528.511

The results also demonstrated high accuracy, as shown in Table 3, with low prediction errors across all racial/ethnic groups. Figure 5e,f summarize the box plot statistics for the 25 states and 196 counties, respectively. In subplots (e) and (f), the correlation measures for different racial and ethnic groups illustrate the performance of the MMC method at the state and county levels. Overall, W (White), B (Black), A (Asian), and H (Hispanic) exhibit relatively stable correlations, while I (Indian and Pacific) and O (Other) show slightly larger variations. Additionally, the correlations at the county level (subplot f) appear slightly lower than those at the state level (subplot e), indicating a minor decrease in predictive stability as the spatial scale becomes more granular. Overall, the method demonstrates high reliability across both levels.

**Table 3:** 10-fold Cross Validation at the County Level.

	W	B	I	A	H	O
Cor	0.999	0.997	0.997	0.992	0.997	0.997
R^2^	0.998	0.994	0.994	0.976	0.994	0.994
MAE	13.093	20.896	21.905	30.449	21.487	21.569
MSE	2050.897	6797.794	6556.244	9575.770	6563.459	6441.301
RMSE	45.287	82.449	80.971	97.856	81.015	80.258
MAPE	0.311	0.289	0.307	0.412	0.290	0.314

To evaluate the accuracy of the ZCTA-level interpolation, we leveraged the Utah Cancer Registry (UCR) dataset—the only available source to the team with reported cancer counts at the ZIP Code Tabulation Area level. This dataset was also used in our prior publication in *Health & Place* and was re-utilized here to ensure consistency and methodological comparability. Specifically, we assessed 3,468 subgroup-ZCTA combinations (289 ZCTAs × 12 population groups), among which 1,156 units contained reported values suitable for validation. A comparison between interpolated and observed values yielded an R^2^ of 0.992, Pearson correlation coefficient of 0.996, and a mean absolute error (MAE) of 3.407. These findings—originally presented in our prior work—are reproduced here to benchmark the public dataset against known ground truth and are visualized in Fig. 5c. We acknowledge that this ZCTA-level validation is limited to a single state due to data availability constraints. However, Utah offers a comprehensive and internally consistent dataset that provides a valuable basis for small-area evaluation. Future work will explore the integration of SEER custom geographic extractions or establish partnerships with additional state cancer registries to expand ZCTA-level validation across the U.S.

The validation results demonstrate that the MMC and Geo-Imputation methods produced reliable and accurate interpolations across all geographic levels. The state- and county-level experiments confirm minimal prediction errors, while the ZCTA-level validation shows high predictive accuracy. These findings underscore the robustness of our methods in handling suppressed and missing cancer incidence data.

## Supplementary information

Supplementary Information

## Data Availability

The code used for generating and processing this dataset is publicly available in **GitHub**: https://github.com/UrbanGISer/UrbanAnalytics/tree/main/Multi_Constraint_Monte_Carlo_GeoImputation. The repository includes all relevant scripts and documentation necessary for reproducing the dataset or adapting the methods to other data sources. **MMC_Simulation.py**: Implements the Multi-Constraint Monte Carlo (MMC) simulation method for imputing suppressed subgroup-specific cancer counts at state and county levels. The script handles all data cleaning, constraint extraction, and iterative simulation processes. **Geo_Imputation.py**: Constructs the Geo-Imputation model to downscale cancer data from state (or county) to ZCTA levels. The script processes demographic data, allocates cancer counts proportionally, and ensures alignment with population distributions. Researchers are encouraged to adapt these scripts to meet specific research objectives. For any questions or technical support, users can refer to the documentation or comment in the repository. The code is compatible with Python (version 3.8 or later) with dependencies outlined in the repository.
