# Supplementary material for: Cancer incidence data at the ZIP Code Tabulation Area level in the United States interpolated by Monte Carlo simulation with multiple constraints
Source: Sci Data. 2025 May 30;12:909. doi: 10.1038/s41597-025-05254-8 (PMC12125315; doi:10.1038/s41597-025-05254-8)
Supplement: Supplementary file 1 — Supplementary Information [file 41597_2025_5254_MOESM1_ESM.docx]

**Supplementary Information**

**Figure S1.** Box plot of interpolated subgroup-specific cancer incidence data for all states in the United States.

**Figure S2.** Results of the Multi-Constraint Monte Carlo simulation for a sample subgroup.

**Figure S3.** Missing values and suppressed cancer incidence data at the state level.

**Table S1.** Evaluation metrics for simulated values compared to observed data across various racial and ethnic groups at the state level.

**Table S2.** Evaluation metrics for simulated values compared to observed data across various racial and ethnic groups at the group level.

**Table S3.** Comparison of evaluation metrics for the Monte Carlo method and machine learning methods.

**Table S4.** FIPS dictionary


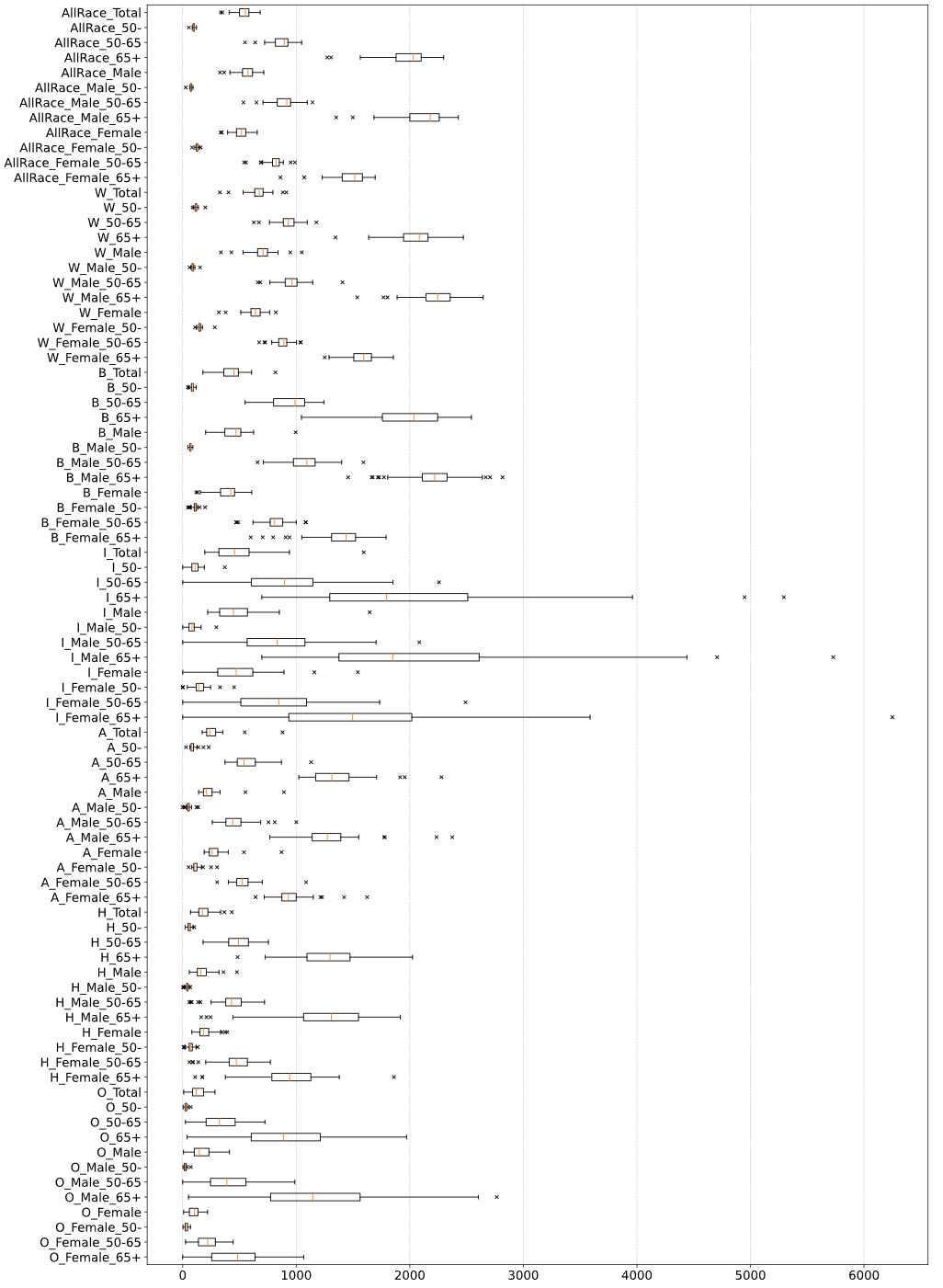


**Figure S1.** Box plot of interpolated subgroup-specific cancer incidence data for all states in the United States.


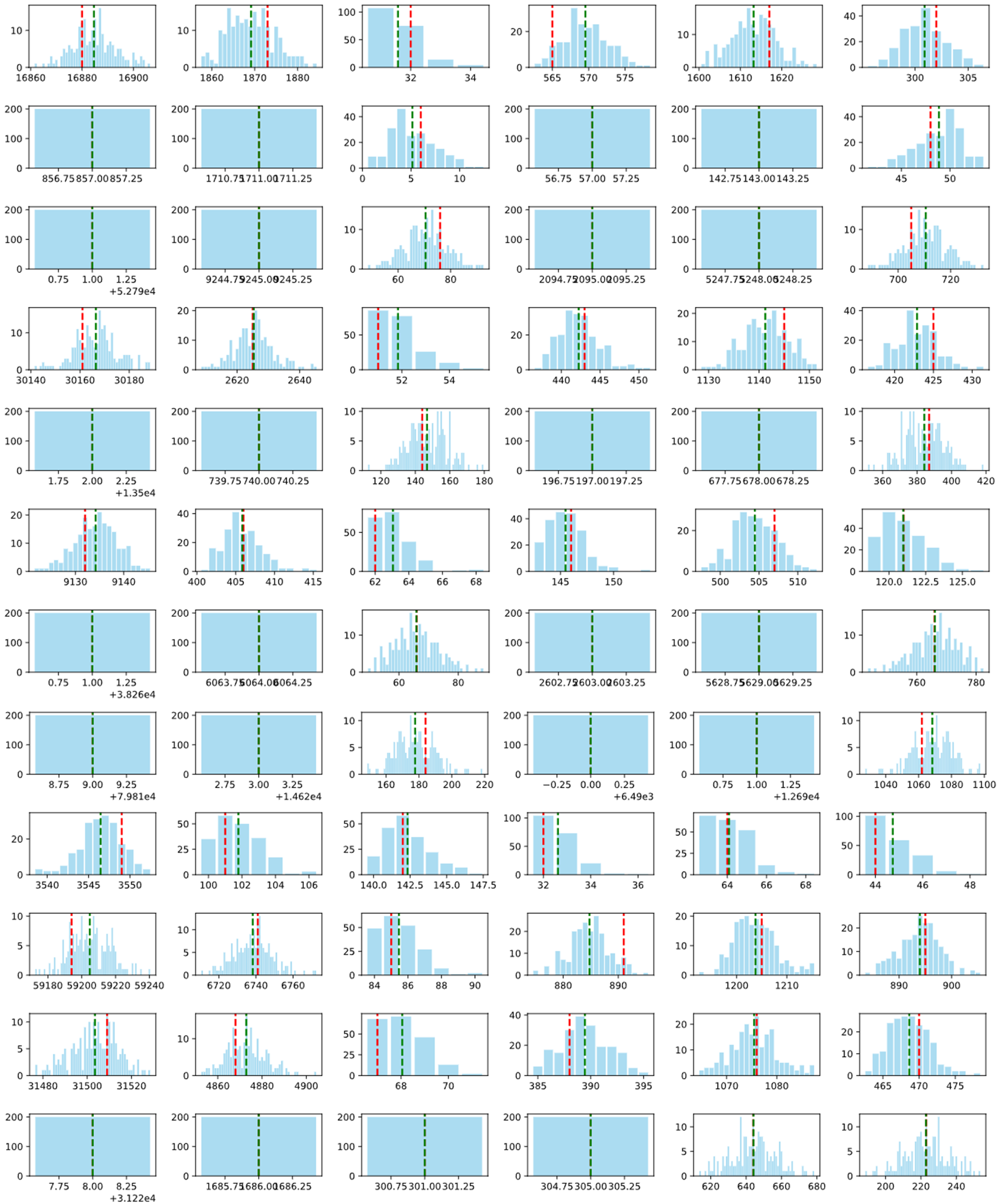


**Figure S2.** Results of the Multi-Constraint Monte Carlo simulation for a sample subgroup.


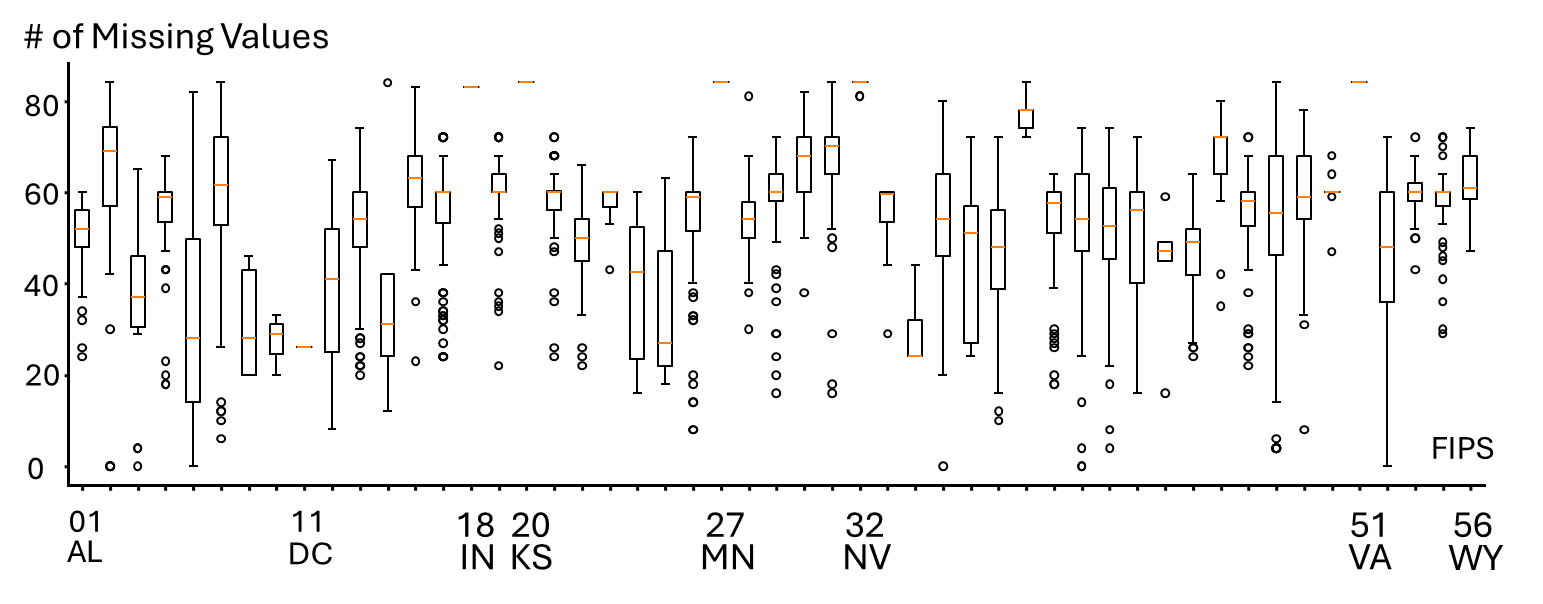


**Figure S3.** Missing values and suppressed cancer incidence data at the state level.

**Table S1.** Evaluation metrics for simulated values compared to observed data across various racial and ethnic groups at the state level.

| Race/  Ethnic | Metrics | Min | Max | Max | SD |
| --- | --- | --- | --- | --- | --- |
| W | MAE | 136.117 | 205.143 | 323.7 | 57.532 |
|  | MSE | 37216.55 | 109074.447 | 283300.967 | 73762.512 |
|  | RMSE | 192.916 | 315.897 | 532.26 | 101.563 |
| B | MAE | 18.14 | 110.826 | 445.867 | 124.021 |
|  | MSE | 597.649 | 117650.28 | 999722.2 | 310503.07 |
|  | RMSE | 24.447 | 209.219 | 999.861 | 286.507 |
| I | MAE | 5.357 | 24.17 | 48.074 | 15.028 |
|  | MSE | 62.607 | 3404.955 | 12900.148 | 4315.495 |
|  | RMSE | 7.912 | 47.51 | 113.579 | 35.711 |
| A | MAE | 43.933 | 97.602 | 170.483 | 48.142 |
|  | MSE | 4797.533 | 53720.91 | 181376.893 | 68825.447 |
|  | RMSE | 69.264 | 191.094 | 425.884 | 138.259 |
| H | MAE | 54.414 | 144.725 | 473.733 | 129.096 |
|  | MSE | 7871.103 | 114576.73 | 774549.267 | 240509.463 |
|  | RMSE | 88.719 | 242.353 | 880.085 | 249.092 |
| O | MAE | 31.207 | 76.676 | 120.982 | 29.938 |
|  | MSE | 2634.414 | 22866.714 | 54377.49 | 19148.299 |
|  | RMSE | 51.327 | 137.905 | 233.19 | 65.395 |

**Table S2.** Evaluation metrics for simulated values compared to observed data across various racial and ethnic groups at the group level.

| Race/  Ethnic | Metrics | Min | Max | Max | SD |
| --- | --- | --- | --- | --- | --- |
| W | MAE | 7.957 | 46.122 | 188.55 | 45.324 |
|  | MSE | 109.283 | 20916.364 | 257508.65 | 52770.389 |
|  | RMSE | 10.454 | 96.845 | 507.453 | 109.627 |
| B | MAE | 1.875 | 24.288 | 71.886 | 21.64 |
|  | MSE | 8.875 | 3479.102 | 19747.086 | 5537.126 |
|  | RMSE | 2.979 | 42.471 | 140.524 | 41.811 |
| I | MAE | 2.0 | 9.924 | 26.304 | 7.823 |
|  | MSE | 4.667 | 340.053 | 1803.174 | 569.115 |
|  | RMSE | 2.16 | 14.088 | 42.464 | 12.621 |
| A | MAE | 0.557 | 7.027 | 51.345 | 11.334 |
|  | MSE | 1.243 | 1054.906 | 18278.745 | 3722.322 |
|  | RMSE | 1.115 | 15.625 | 135.199 | 29.061 |
| H | MAE | 2.833 | 19.958 | 87.077 | 25.485 |
|  | MSE | 9.167 | 3264.412 | 33086.117 | 8194.755 |
|  | RMSE | 3.028 | 32.676 | 181.896 | 47.972 |
| O | MAE | 4.2 | 16.958 | 57.357 | 17.701 |
|  | MSE | 22.2 | 993.889 | 7001.929 | 2176.193 |
|  | RMSE | 4.712 | 20.961 | 83.678 | 24.822 |

**Table S3.** Comparison of evaluation metrics for the Monte Carlo method and machine learning methods.

| Metrics | MC | ML with Population | | | | | ML with population  and ancillary data | | | | |
| --- | --- | --- | --- | --- | --- | --- | --- | --- | --- | --- | --- |
|  |  | GLM | GBM | RF | XGB | OLS | GLM | GBM | RF | XGB | OLS |
| MAE | 3.41 | 124.16 | **12.28** | 12.36 | 10.08 | 27.98 | 132.97 | **14.76** | 178.62 | 23.83 | 83.52 |
| MSE | 27.61 | 25770 | 3825 | 7996 | **328** | 1382 | 31676 | **559** | 45733 | 1572 | 10050 |
| RMSE | 5.25 | 160.5 | 61.9 | 89.4 | **18.1** | 37.2 | 178.0 | **23.7** | 213.9 | 39.6 | 100.3 |
| MAPE | 0.17 | 8.44 | 0.34 | **0.30** | 0.40 | 2.26 | 8.53 | **0.93** | 13.89 | 1.24 | 6.66 |
| R | 0.991 | 0.60 | 0.61 | 0.48 | **0.91** | 0.83 | 0.59 | **0.84** | 0.43 | 0.63 | 0.43 |
| R2 | 0.98 | 0.36 | 0.37 | 0.23 | **0.83** | 0.69 | 0.35 | **0.71** | 0.18 | 0.40 | 0.18 |

Note: MAE: mean absolute error, MSE: mean squared error, RMSE: root mean squared error, MAPE：mean absolute percentage error, R: Correlation, R^2^: Squared R. Values in **bold font** indicate the best-performing ML models.

**Table S4.** FIPS dictionary.

01 ALABAMA

02 ALASKA

04 ARIZONA

05 ARKANSAS

06 CALIFORNIA

08 COLORADO

09 CONNECTICUT

10 DELAWARE

11 DISTRICT OF COLUMBIA

12 FLORIDA

13 GEORGIA

15 HAWAII

16 IDAHO

17 ILLINOIS

18 INDIANA

19 IOWA

20 KANSAS

21 KENTUCKY

22 LOUISIANA

23 MAINE

24 MARYLAND

25 MASSACHUSETTS

26 MICHIGAN

27 MINNESOTA

28 MISSISSIPPI

29 MISSOURI

30 MONTANA

31 NEBRASKA

32 NEVADA

33 NEW HAMPSHIRE

34 NEW JERSEY

35 NEW MEXICO

36 NEW YORK

37 NORTH CAROLINA

38 NORTH DAKOTA

39 OHIO

40 OKLAHOMA

41 OREGON

42 PENNSYLVANIA

44 RHODE ISLAND

45 SOUTH CAROLINA

46 SOUTH DAKOTA

47 TENNESSEE

48 TEXAS

49 UTAH

50 VERMONT

51 VIRGINIA

53 WASHINGTON

54 WEST VIRGINIA

55 WISCONSIN

56 WYOMING
